# “Internet+Nursing Service” Mobile Apps in China App Stores: Functionality and Quality Assessment Study

**DOI:** 10.2196/52169

**Published:** 2024-02-16

**Authors:** Shuo Yuan, Min Liu, Yuqi Peng, Jinrui Hu, Bingyan Li, Xia Ding, Lunfang Xie

**Affiliations:** 1 School of Nursing, Anhui Medical University, Hefei, China; 2 Department of Cardiology II, Anhui No.2 Provincial People’s Hospital, Hefei, China; 3 Nursing Department, Anhui No.2 Provincial People’s Hospital, Hefei, China

**Keywords:** mobile phone, mobile applications, home care services, telemedicine, nursing services, China, apps

## Abstract

**Background:**

As the Chinese society ages and the concern for health and quality of life grows, the demand for care services in China is increasing. The widespread use of internet technology has greatly improved the convenience and efficiency of web-based services. As a result, the Chinese government has been implementing “Internet+Nursing Services” since 2019, with mobile apps being the primary tools for users to access these services. The quality of these apps is closely related to user experience and the smooth use of services.

**Objective:**

This study aims to evaluate the functionality, services, and quality of “Internet+Nursing Service” apps; identify weaknesses; and provide suggestions for improving service programs and the research, development, improvement, and maintenance of similar apps.

**Methods:**

In December 2022, two researchers searched for “Internet+Nursing Service” apps by applying the search criteria on the Kuchuan mobile app monitoring platform. After identifying the apps to be included based on ranking criteria, they collected information such as the app developer, app size, version number, number of downloads, user ratings, and number and names of services. Afterward, 5 trained researchers independently evaluated the quality of the apps by using the Chinese version of the user version of the Mobile App Rating Scale (uMARS-C). The total uMARS-C score was based on the average of the five evaluators’ ratings.

**Results:**

A total of 17 “Internet+Nursing Service” apps were included. Among these, 12 (71%) had been downloaded more than 10,000 times, 11 (65%) had user ratings of 4 or higher, the median app size was 62.67 (range 22.71‐103; IQR 37.51-73.47) MB, 16 (94%) apps provided surgical wound dressing change services, 4 (24%) covered first-tier cities, and only 1 (6%) covered fourth-tier cities. The median total uMARS-C score was 3.88 (range 1.92-4.92; IQR 3.71-4.05), which did not correlate with app store user ratings (*r*=0.003; *P*=.99). The quality of most apps (11/17, 65%) was average. Most apps (12/17, 71%) were rated as “good” or above (≥4 points) in terms of information quality, layout, graphics, performance, and ease of use; however, the vast majority of apps were rated as “fair” or even “poor” (<4 points) in terms of credibility (14/17, 82%) and demand (16/17, 94%).

**Conclusions:**

“Internet+Nursing Service” apps need to broaden their service coverage, increase service variety, and further optimize their service structure. The overall quality of these apps is generally poor. App developers should collaborate with medical professionals and communicate with target users before launching their products to ensure accurate content, complete functionality, and good operation that meets user needs.

## Introduction

As IT continues to develop, the number of internet users worldwide has reached 4.95 billion in 2022, including 1.067 billion internet users in China, with an internet penetration rate of 75.6%. The proportion of mobile phone users accessing the internet has reached 99.6% [1]. Mobile apps continue to grow steadily, with people using them for communication, shopping, office work, and socializing. The field of internet health care is becoming increasingly standardized, and it was the fastest-growing field in terms of user scale in 2022. As of December 2022, the number of internet health care users in China reached 363 million—an increase of 64.66 million when compared to December 2021—accounting for 34% of all internet users [1]. Mobile health (mHealth) apps are the main media for mHealth and the primary channels for users to obtain medical support and health information. In 2019, 65.9% of internet users chose medical and health apps for medical consultations when feeling unwell [2].

People are increasingly inclined to use health care apps to manage their health due to these apps’ cost-effectiveness, convenience, and speed in accessing health information [3-5] and ability to provide evidence-based health information for better health guidance [6]. There are several popular health care apps in China. According to a report on Statista, as of December 2022, the most popular medical app in China was Ping An Good Doctor—a health care platform owned by the Ping An Insurance Group—with almost 23 million monthly active users [7]. The mobile portal provides real-time medical consultations, web-based appointment booking services, and a health-related discussion forum.

As people become more health conscious, the demand for health care services is increasing. To alleviate the pressure on offline medical institutions and meet the needs of the public, China launched “Internet+Nursing Services” [8]. These services are provided by registered nurses from fixed medical institutions and operate on a web-based app and offline service model. They are designed to serve discharged patients or special populations with medical conditions and limited mobility [9]. Since 2019, “Internet+Nursing Service” organizations in China’s provinces and regions have been actively providing services to countless older adults living at home, pregnant women, infants, and young children who have difficulty with leaving their homes. These services have received unanimous positive reviews [10,11]. The “Internet+Nursing Service” model integrates nursing services with internet technology, using internet IT to break the spatial limitations of traditional medical treatment. It can meet the multilevel nursing care needs of service users, allow for personalized and continuous care, alleviate social problems that result in difficulties with visiting a physician, improve the quality of life of service users, and broaden the channels of communication between nurses and patients [12].

Patients place orders through an app, and managers dispatch orders based on various factors, such as web-based nurses’ qualifications, professionalism, and distance. Platform nurses receive orders within a specified period of time and travel to patients’ homes to provide services, including routine nursing operations (eg, intramuscular injections, intravenous injections, urinary catheterization, gastric tube insertion, and blood sample collection), as well as specialty care (eg, peripherally inserted central venous catheter medication exchange, wound stoma care, and neonatal examinations). By downloading high-quality “Internet+Nursing Service” mobile apps, users can quickly access the care services they need without leaving their homes. However, low-quality apps may not only affect the user experience but also make it impossible for users to obtain the care services they need.

As people become increasingly dependent on smartphones and apps, they are also becoming more concerned about the quality of apps. Users not only expect apps to function properly but also have high expectations for their aesthetics, security, and personalization settings [13]. App quality issues can affect the user experience, determine whether users continue to use the app, and even lead to economic and property losses for both the users and the app development departments. mHealth apps are a special type of app, and studies have shown that factors such as usability, navigability, accuracy of information, and security all affect the user’s experience with and evaluation of an mHealth app. If an mHealth app is of low quality, users may doubt its usefulness and effectiveness, which can even lead to users obtaining low-quality health services and generating erroneous health management concepts that affect their health beliefs and behaviors [6,14,15].

In recent years, various software development organizations have been paying more attention to improving app quality, but there are still deficiencies [16]. The quality of an app is mainly judged by checking user ratings and reviews on app stores. However, the actual quality of an app can be unclear, and it is impossible to know whether an app’s functions are comprehensive or whether its content is scientific based on the data displayed on app store pages. Therefore, it is necessary to objectively evaluate the quality of medical and health apps to identify shortcomings in their development; promote their continuous improvement; improve their applicability, usage experience, and compliance (eg, compliance with data protection laws); and allow users to use apps that are of high quality, are reasonably designed, and are safe to use. There are various methods for evaluating the quality of medical apps, which are also known as *mHealth apps*. According to a study by Stoyanov et al [17], the quality of mHealth apps is evaluated based on different categories, including engagement, functionality, aesthetics, information quality, and subjective quality [18]. There are various methods for evaluating mHealth services, such as the use of questionnaires, the conduction of interviews, and observation [18]. A systematic review by Nouri et al [19] identified the following seven main classes of assessment criteria for mHealth apps: design, information and content, usability, functionality, ethical issues, security and privacy, and user-perceived value. These criteria can be used to assess the quality of a medical app.

Most studies on “Internet+Nursing Services” focus on service effects [20-22], the establishment of service quality index systems [23], risk management strategies [24,25], and the demand for services from various groups [26-28]. However, there is little attention paid to the quality of “Internet+Nursing Service” apps and a lack of studies that use evaluation tools to objectively evaluate these apps’ quality and functions. The aim of this study was to review the “Internet+Nursing Service” mobile apps that are available on China’s app stores and evaluate their quality.

## Methods

### Selection of the “Internet+Nursing Service” Apps

We used the Kuchuan mobile app monitoring platform (Beijing Kuchuan Technology Co.) to monitor data from the iOS and Android app stores. This platform provides real-time information about mobile app developers, the latest versions of apps, and the number of app downloads. Two researchers searched for apps that were available as of December 1, 2022, using the keywords “Internet+Nursing Service,” “Home Nursing,” “Nurses at Home,” “online nurse,” and “shared nurse.”

The inclusion criteria for the apps were (1) apps with content that includes home nursing services, (2) apps categorized as health care apps, (3) apps in Chinese, (4) free apps, and (5) functional apps. The exclusion criteria were (1) non–user-side apps, (2) old versions of the same app, (3) apps with different names but the same content, and (4) duplicate apps.

**Figure 1.**
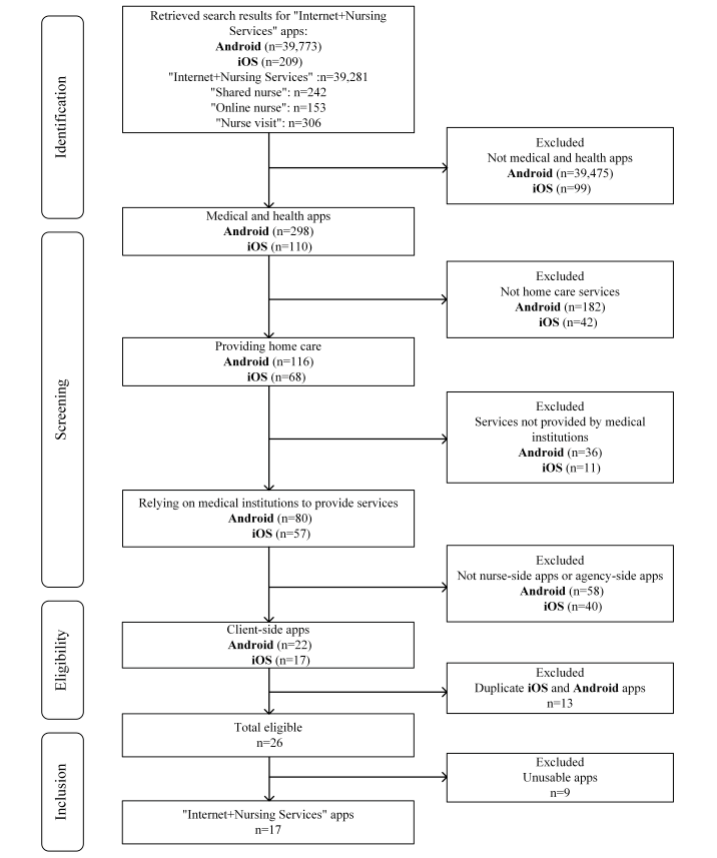
Flowchart of the selection of apps.

Two researchers independently screened the apps based on their names, profiles, and display images. They then discussed their findings to finalize the list of evaluated apps (Figure 1).

### Selection of a Standardized Rating Scale for Mobile Apps

We used the Chinese version of the user version of the Mobile App Rating Scale (uMARS-C), and we obtained authorization from the authors of the uMARS-C [29]. Adapted from the Mobile App Rating Scale (MARS), the user version of the MARS (uMARS) has been used for assessing a wide variety of apps, including apps for mental health [30], rheumatology patient management [31], cancer risk assessment [32], and hospital registration [33]. The uMARS-C includes 14 objective items that are rated on a 5-point Likert scale and divided into the following three dimensions: engagement, functionality, and information. Dimension scores are calculated by dividing the total entry score by the number of entries, and the uMARS-C total score is calculated by dividing the total dimension score by the number of dimensions. According to the rating scale, a uMARS-C total score of 1 indicates poor quality, a score of 2 indicates inadequate quality, a score of 3 indicates fair quality, a score of 4 indicates good quality, and a score of 5 indicates excellent quality.

The uMARS-C has good reliability and validity, with a Cronbach α coefficient of 0.890 and dimension Cronbach α coefficients ranging from 0.853 to 0.895. The test-retest reliability value is 0.967, the item content validity index ranges from 0.78 to 1.00, and the scale content validity index/average is 0.969.

### Process of Evaluating “Internet+Nursing Service” Apps

A total of 5 researchers—2 nurses with more than 5 years of experience in their roles, 2 graduate nursing students, and 1 professional internet engineer with more than 7 years of experience in their role—assessed the quality of the apps. Before the evaluation, we made sure that each researcher properly understood and was familiar with the uMARS-C. To ensure their understanding of the scale, they downloaded and assessed 2 mHealth apps that were not included in this study. When there was a difference of more than 2 points in dimension scores or total scores, they discussed until reaching a consensus. The researchers then downloaded the apps that were included in the final analysis onto iOS and Android smartphones. After downloading the apps, they used each app for at least 10 minutes and independently evaluated the ease of use, performance, security, and settings of each app, using the uMARS-C. Basic app information was collected from the app store download page, including the app developer, app size (in MB), version number, number of downloads, user ratings (ranging from 0 to 5), and number and names of services, among others. Some app download data were missing because the iOS app store did not provide these data. The researchers also graded the service coverage cities based on the categorized statistical service items in the *Beijing Internet Home Care Service Item Catalog* (2022 edition) [34] and the city class divisions in the *2022 China’s City Business Attractiveness Ranking* [35].

### Statistical Analysis

We used EpiData 3.1 (EpiData Association) for data entry and SPSS 24.0 (IBM Corp) for statistical analysis. Nonnormally distributed measurement data were expressed as medians and quartiles, while count data were expressed as numbers and percentages. The uMARS-C dimension scores for each app were averaged across the five raters, and the final scores were calculated by using the scale’s formula.

### Ethical Considerations

This study did not involve human subjects, clinical trials, and vulnerable groups and was therefore exempt from ethical approval.

## Results

### Characteristics of Selected Apps

Our search found a total of 39,982 apps (iOS: n=209; Android: n=39,773). After initial screening based on the inclusion and exclusion criteria, we downloaded 26 apps. After using them, we excluded 8 apps that were not working properly, leaving a total of 17 apps, which were included in this study (Table 1 and Multimedia Appendix 1). Of these 17 apps, 4 (24%) were released by medical institutions and 13 (76%) were released by corporations (Table 1). Further, 12 (71%) apps had been downloaded more than 10,000 times, with Champion Nurse having the highest number of downloads (n=37,321,776). The median app size was 62.67 (range 22.71-103; IQR 37.51-73.47) MB. App store user ratings ranged from 2.8 to 5.0, with 14 (82%) apps being rated 3.0 or higher and 11 (65%) apps being rated 4.0 or higher. In terms of service coverage, 4 (24%) of the “Internet+Nursing Service” apps covered first-tier cities, including Beijing; 8 (47%) covered new first-tier cities; 4 (24%) covered second-tier cities; and 1 (6%) covered fourth-tier cities.

**Table 1.** Characteristics of the “Internet+Nursing Service” apps.

Characteristic		Apps (N=17), n (%)
**Download count** ^**a**^		
	0-9999	2 (12)
	10,000-99,999	7 (41)
	100,000-999,999	3 (18)
	1,000,000-9,999,999	1 (6)
	≥10,000,000	1 (6)
**Platform**		
	iOS and Android	13 (76)
	iOS	3 (18)
	Android	1 (6)
**User rating (number of stars)**		
	0-2.9	3 (18)
	3.0-3.9	3 (18)
	4.0-4.9	5 (29)
	5.0	6 (35)
**Developer**		
	Individual developer	8 (47)
	Corporation	9 (53)
**Number of services provided**		
	1-10	2 (12)
	11-20	9 (53)
	21-30	6 (35)
**Service coverage city class**		
	First-tier cities	4 (24)
	New first-tier cities	8 (47)
	Second-tier cities	4 (24)
	Fourth-tier cities	1 (6)

a Download count for Android apps only.

### Categories of Nursing Services Provided by Apps

The 17 apps provided at-home nursing services, including intravenous injection, intramuscular injection, and nebulized inhalation services, among others. These services were classified as *Health Assessment and Guidance*, *Clinical Nursing*, *Maternal and Infant Nursing*, *TCM* (traditional Chinese medicine) *Nursing*, *Specialty Nursing*, *Hospice*, and *Rehabilitation Nursing* services per the categories in the *Beijing Internet Home Care Service Item Catalog* [34]. Any services that were not in the catalog were classified as *uncategorized items*.

Of the 17 apps, 16 (94%) provided surgical wound dressing change services (*Clinical Nursing* category), 15 (88%) provided services for the maintenance of peripherally inserted central catheters (*Specialty Nursing* category), 14 (82%) provided maternal and infant nursing services (*Maternal and Infant Nursing* category), and 10 (59%) provided TCM nursing services (*TCM Nursing* category) and disease rehabilitation guidance (*Rehabilitation Nursing* category). Further, 4 (24%) apps provided hospice care services (*Hospice* category), 3 (18%) provided health assessments and guidance (*Health Assessment and Guidance* category), and only 1 (6%) app provided gastrointestinal decompression, previsit physical examination, conjunctival capsule irrigation, T-tube drainage care, and family room services (uncategorized items).

### Quality of the “Internet+Nursing Service” Apps

**Figure 2.**
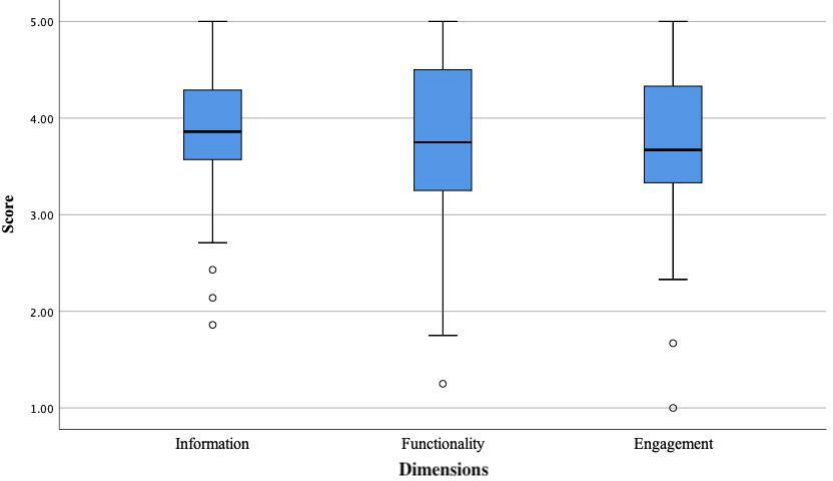
uMARS-C overall scores of the “Internet+Nursing Service” apps (N=17). The bottom and top edges of the boxes represent the first and third quartiles, respectively; the lines within the boxes represent the medians; the ends of the bottom and top whiskers represent the minimum and maximum values, respectively; and the circles represent outliers. uMARS-C: Chinese version of the user version of the Mobile App Rating Scale.

**Figure 3.**
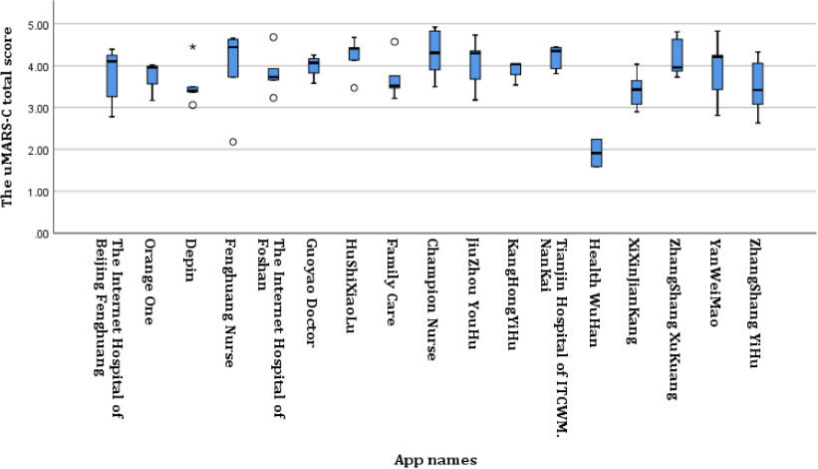
uMARS-C dimension scores of “Internet+Nursing Service” apps (N=17). The bottom and top edges of the boxes represent the first and third quartiles, respectively; the lines within the boxes represent the medians; the ends of the bottom and top whiskers represent the minimum and maximum values, respectively; the circles represent outliers; and the asterisk represents an extreme outlier. uMARS-C: Chinese version of the user version of the Mobile App Rating Scale.

In our study, the Cronbach α coefficient of the uMARS-C was 0.871, and the dimension Cronbach α coefficients ranged from 0.761 to 0.811. Based on the uMARS-C scores, of the 17 apps, 1 (6%) was rated as “poor,” 11 (65%) were rated as “fair,” 5 (29%) were rated as “good,” and none were rated as “insufficient” or “excellent.” The median total score for the “Internet+Nursing Service” apps was 3.88 (range 1.92-4.92; IQR 3.71-4.05; Figure 2), with Champion Nurse having the highest score (4.92) and Health WuHan having the lowest score (1.92). There was no significant correlation between app store user ratings and total uMARS-C scores (*r*=0.003; *P*=.99). The median information dimension score was 3.97 (range 1.86-5.00; IQR 3.57-4.29), the median functionality dimension score was 3.95 (range 1.25-5.00; IQR 3.25-4.50), and the median engagement dimension score was 3.80 (range 1.00-5.00; IQR 3.33-4.33; Figure 3). A heat map comparing the scores of each entry in the uMARS-C for the 17 “Internet+Nursing Service” apps showed that Health WuHan scored below 3 points in most entries (11/14, 79%; Multimedia Appendix 2). Further, 14 (82%) apps scored below 4.0 points in the *Credibility* entry, indicating an average or poor level; 16 (94%) scored below 4.0 points in the *Demand* entry; 14 (82%) scored at a good or above level in the *Quality of Information* entry; and 12 (71%) scored at a good or above level in the *Layout*, *Graphics*, *Performance*, and *Ease of Use* entries.

## Discussion

### Principal Results

As internet technology continues to develop, all industries are integrating the “Internet+” model to promote innovation and development [1]. However, unlike services in other industries, “Internet+Nursing Services,” as web-based health care services, are characterized by the high risks and high professionalism of the medical industry, as well as the special risks associated with mHealth [25].

There are several risks associated with “Internet+Nursing Service” apps. One of the risks is that technology barriers can prevent some patients from accessing telehealth services. These barriers can include a lack of access to the internet, a lack of access to the necessary devices, or difficulty with using the technology. Another risk is that there may be issues with insurance coverage for telehealth services, as well as regulatory obstacles that can limit the use of telehealth [36]. Therefore, it is necessary to strengthen the supervision of “Internet+Nursing Service” apps and strictly control all aspects. “Internet+Nursing Services” are human-centered services and aim to improve health. Therefore, “Internet+Nursing Service” app design should fully consider user characteristics and needs. In this study, we downloaded and used “Internet+Nursing Service” apps, evaluated them from a user perspective, and rated them objectively based on our usage experience.

Most apps (16/17, 94%) provided services in first-tier cities, new first-tier cities, or second-tier cities, with only 1 app providing “Internet+Nursing Services” in a fourth-tier city. The “Internet+Nursing Service” scope does not yet cover remote areas and townships. Data from China’s seventh national census show that the rural population consists of about 509.79 million people, accounting for 36.11% of the total population [37]. However, 80% of medical resources are concentrated in medium- and large-sized medical institutions in medium- and large-sized cities, leading to an imbalance between the demand for care and the supply of care resources for the grassroots population [38].

Providing high-quality nursing resources and services is key to improving the health and quality of life of people at the grassroots level. Medical institutions at all levels should actively promote the distribution of medical resources to enhance access to basic medical and public health services. Therefore, “Internet+Nursing Service” apps should integrate medical resources and promote a 3-tier “hospital-community-family” linkage to bring professional nursing services into the homes of grassroots people. This would encourage more medical institutions to provide home care, expand the scope of services, make full use of medical resources, and address the imbalance between the supply of and demand for medical resources.

Our study found that “Internet+Nursing Service” apps provide a limited number of service programs—mostly routine care programs—with few special care programs, such as psychological care, hospice care, and child care programs. Only 4 of the 17 apps provided hospice care services, and none provided psychological care services, despite the high demand for these programs. In one study, it was found that 92.3% of the older adult population in urban and rural areas needed psychological comfort [39], and in another study, 10.32% of housebound older adults believed that hospice care should be carried out [40]. Further, as the concept of childbearing changes, people are pursuing more scientific and specialized childcare, and child health care and nursing have become more emphasized. Research has shown that providing nutritional guidance, growth and development guidance, and child psychological care to families of preterm infants through “Internet+Nursing Service” platforms could promote the growth and intellectual development of preterm infants [41]. Medical institutions in each region should have an in-depth understanding of the needs of service users and the characteristics of different groups of people. They should gradually expand the list of “Internet+Nursing Services” by taking into account the actual situations of medical institutions and the local medical resources to optimize the structure of service items and meet the needs of service users.

The total quality scores of the 17 apps ranged from 1.92 to 4.92, with 1 (6%) app rated as “poor” and 11 (65%) rated as “fair,” indicating that the overall quality of “Internet+Nursing Service” apps was not good. This may be related to the fact that app development engineers do not fully understand the medical industry. Most “Internet+Nursing Service” apps (9/17, 53%) were developed by corporations, and the developers may not have taken into account the specificity of medical software before development. They also may not have communicated well with medical staff during app development or understood the content and characteristics of “Internet+Nursing Service” apps.

The process of target user evaluation not only strengthens the interactions between users and the software but also identifies weak points (ie, from user feedback) that developers may have missed [42]. Our researchers found that some apps had problems, such as crashes and the inability to log in during use, which affected the user experience. Therefore, research and development organizations need to conduct premarket research and postmaintenance work to ensure the smooth operation of platforms.

From the heat map (Multimedia Appendix 2), it can be seen that most apps (12/17, 71%) scored at a good or above level in terms of the *Layout*, *Graphics*, *Performance*, and *Ease of Use* entries. This indicates that most app development teams pay more attention to the visual effects, ease of use, and smoothness of their apps. However, of the 17 apps, 14 (82%) had average scores of less than 4.0 in the *Credibility* entry, and 16 (94%) had average scores of less than 4.0 in the *Demand* entry, indicating average or even poor performance in these areas. During the evaluation, our researchers found that some app development teams did not clearly label the source or publisher information when publishing health science articles or videos. This may cause users to doubt the authenticity, authority, and reliability of the articles when reading them, affecting their ability to build health knowledge and manage their own health [43]. It is important to publish health science articles with scientific evidence and to clarify the sources, authors, time of publication, and applicable populations of standardized content to increase the credibility of information. In terms of meeting user needs, we found that most apps provided information from health science literature in text form, with content mostly focused on introducing services, medical institutions, and health care experts. We also noted that after content was published on an app, the content was not updated for a long time. Some apps also only had 2 to 3 articles and could not meet user needs. Therefore, in addition to not meeting user needs in terms of service programs, there are also deficiencies in providing up-to-date and relevant health information.

App developers can improve the credibility of health science articles and videos by taking several steps. First, they should ensure that the information provided is based on scientific evidence and comes from reputable sources. This can be done by clearly labeling the sources, authors, time of publication, and applicable populations of the content. Second, they should regularly update the content to ensure that it is current and relevant. Third, they should provide references or links to the original sources of information, so that users can verify the accuracy of the information. By taking these steps, app developers can increase the credibility of their health science articles and videos and help users build their health knowledge and manage their own health.

Health science popularization should aim to provide basic concepts and knowledge in the field of health, with a focus on healthy lifestyles and behaviors. Health science content should be regularly updated to keep up with social hot spots, seasonal changes, and the occurrence of epidemics to provide users with the most up-to-date and relevant information. To achieve this, app development teams should conduct market research to understand the needs and characteristics of their users, including users’ cultural levels and reading habits. They should also keep track of social hot spots, seasonal changes, and other factors to provide relevant health policies, basic medical knowledge, diet and exercise guidance, psychological guidance, and knowledge regarding disease prevention or first aid in daily life. In addition to providing information in graphic form, app developers can also use video and audio formats to present health science information from multiple angles, dimensions, and levels to meet the needs of users at different levels. By providing accurate and scientific health information in a timely manner, app developers can help users improve their health knowledge and quality of life.

Our study found no correlation between uMARS-C ratings and app store ratings, suggesting that app store ratings do not reflect the quality of apps. This may have been due to the small number of app store ratings, differences in app store rating mechanisms, developer marketing strategies, or users’ preferences (eg, app favoritism among users). If the quality of apps is judged solely based on app store ratings, users may download low-quality apps, thereby affecting their usage experience and even causing them to distrust care services. Additionally, health care professionals may be unable to accurately recommend high-quality apps to patients or their families. This further demonstrates the necessity of evaluating the quality of “Internet+Nursing Service” apps by using objective rating scales.

### Limitations

This study has several limitations. First, we only searched for “Internet+Nursing Service” apps that were updated until December 1, 2022, and did not consistently track the uploads and downloads of related apps. Second, we only used iPhone, Huawei, and Xiaomi phones to download and evaluate apps and did not use other systems, such as Meizu, Samsung, and Windows phones. During app development, the development team may modify app functions for different systems due to differences in system algorithms, resulting in differences in app functions. Therefore, future research should take system differences into account and conduct more comprehensive quality evaluations of apps for different systems. Third, there were only 5 researchers in this study; all were under the age of 30 years and had a high level of e-literacy, which may have introduced bias in entries, such as the *Ease of Use* entry, during the evaluation process. However, older people, who are the main target of “Internet+Nursing Services,” have varying levels of e-literacy and may have different understandings and judgments of an app’s ease of use. Therefore, future studies may consider including evaluators with different backgrounds and health literacy levels.

### Conclusions

In this study, we used the uMARS-C to evaluate the quality of “Internet+Nursing Service” apps. We found that the service coverage of these apps was concentrated in first-tier cities (eg, Beijing and Shanghai), new first-tier cities, and some second-tier cities, with a limited number of service items and a need to optimize the structure of service items. The quality evaluation results showed that the quality of apps was not good, especially in terms of information credibility and meeting users’ needs. Further, the scale scores did not correlate with app store scores. Therefore, “Internet+Nursing Service” app development teams need to pay attention to improving the quality of their apps. Before releasing an “Internet+Nursing Service” app, they should fully understand the needs of their target users, as well as the characteristics of this type of app, and communicate with relevant professionals in the field. They should also orient the release of health knowledge in the app toward user needs and improve the credibility and readability of content. After releasing an app, it is necessary to maintain and update it to ensure its normal operation and the timely updating of health education content.

## Supplementary material

10.2196/52169 Multimedia Appendix 1 Details of the 17 “Internet+Nursing Service” apps.

10.2196/52169 Multimedia Appendix 2 Heat map of the average scores for each item and app. The colors range from blue (worst score) to white (best score).

## Abbreviations

MARS: Mobile App Rating Scale
mHealth: mobile health
TCM: traditional Chinese medicine
uMARS: user version of the Mobile App Rating Scale
uMARS-C: Chinese version of the user version of the Mobile App Rating Scale
