# Supplementary material for: “Internet+Nursing Service” Mobile Apps in China App Stores: Functionality and Quality Assessment Study
Source: JMIR Mhealth Uhealth. 2024 Feb 16;12:e52169. doi: 10.2196/52169 (PMC10912935; doi:10.2196/52169)
Supplement: Multimedia Appendix 1 [file mhealth-v12-e52169-s001.doc]

| **App** **name** | **Download count** | **Latest version** | **User**  **rating** | **App**  **size**  **(MB)** | **Developer** | **Platform** | **Number of**  **services**  **provided** | **Service coverage cities** | **total**  **uMARS-C score** |
| --- | --- | --- | --- | --- | --- | --- | --- | --- | --- |
| The Internet  Hospital of Beijing  Fenghuang | 10223 | 1.0.7 | 5 | 85.4 | Medical institution | IOS＆ Android | 14 | Beijing | 3.75 |
|  |  |  |  |  |  |  |  | Beijing, Xiamen, Guangzhou, |  |
|  |  |  |  |  |  |  |  | Chengde, Wuhan, Nanjing, |  |
| Orange One | / | 1.0.5 | 5 | 70.2 | Corporation | IOS | 11 | Changzhou, Wuxi, Jinan, Shanghai, Chengdu, Hangzhou, Wenzhou, Ningbo, Chongqing | 3.72 |
| Depin | 10011 | 1. 1.6 | 5 | 23. 1 | Corporation | IOS＆ Android | 16 | Nanjing | 3.56 |
| Fenghuang Nurse | 12233 | 1.4.0 | 4.5 | 61.6 | Corporation | IOS＆ Android | 19 | Beijing | 3.93 |
| The Internet Hospital of Foshan | 462515 | 1.8.5 | 3 | 64. 1 | Medical institution | IOS＆ Android | 18 | Foshan | 3.85 |
|  |  |  |  |  |  |  |  | Hubei, Shanxi, Henan, Inner |  |
| Guoyao Doctor | / | 78.0.0 | 4.5 | 103 | Corporation | IOS | 21 | Mongolia, Heilongjiang,  Anhui, Liaoning | 3.97 |
| HuShiXiaoLu | 887823 | 2.5.9 | 5 | 24.7 | Corporation | IOS＆ Android | 29 | Hangzhou | 4.22 |
| Family Care | 20323 | 2. 12.3 | 4.2 | 37.51 | Corporation | IOS＆ Android | 18 | Binzhou | 3.71 |

| **App** **name** | **Download count** | **Latest version** | **User**  **rating** | **App**  **size**  (MB) | **Developer** | **Platform** | **Number of**  **services**  **provided** | **Service coverage cities** | **total**  **uMARS-C score** |
| --- | --- | --- | --- | --- | --- | --- | --- | --- | --- |
| Health WuHan | 165731 | 4.3.0 | 2.9 | 27.9 | Corporation | Android | 8 | Wuhan | 1.92 |
| Champion Nurse | 37321776 | 4.7.2 | 3.8 | 99.8 | Corporation | IOS＆ Android | 27 | Beijing | 4.29 |
| JiuZhou YouHu | 72510 | 3.1.0 | 4.8 | 56.1 | Corporation | IOS＆ Android | 30 | Weihai | 4.05 |
| KangHongYiHu | / | 2.0.5 | 5 | 62.67 | Corporation | IOS | 22 | Qingdao | 3.88 |
| Tianjin Hospitall  of  ITCWM ·NanKai | 3547 | 1.0.10 | 2.8 | 44.45 | Medical institution | IOS＆ Android | 1 | Tianjing | 4. 19 |
| XiXinJianKang | 6132595 | 3.17.3 | 2.9 | 96.57 | Corporation | IOS＆ Android | 18 | Shenyang, Liaoyang,Ningbo, Xiaogan | 3.42 |
| YanWeiMao | 10657 | 1.5.0 | 5 | 22.71 | Corporation | IOS＆ Android | 23 | Xian | 3.91 |
| ZhangShang XuKuang | 84040 | 2.0.1 | 3.7 | 70.1 | Medical institution | IOS＆ Android | 15 | Xuzhou | 4.20 |
| ZhangShang YiHu | 2614 | 1.6.6 | 4.9 | 73.47 | Corporation | IOS＆ Android | 17 | Hunan | 3.50 |
