# Supplementary figures and images for: “Internet+Nursing Service” Mobile Apps in China App Stores: Functionality and Quality Assessment Study
Source: JMIR Mhealth Uhealth. 2024 Feb 16;12:e52169. doi: 10.2196/52169 (PMC10912935; doi:10.2196/52169)

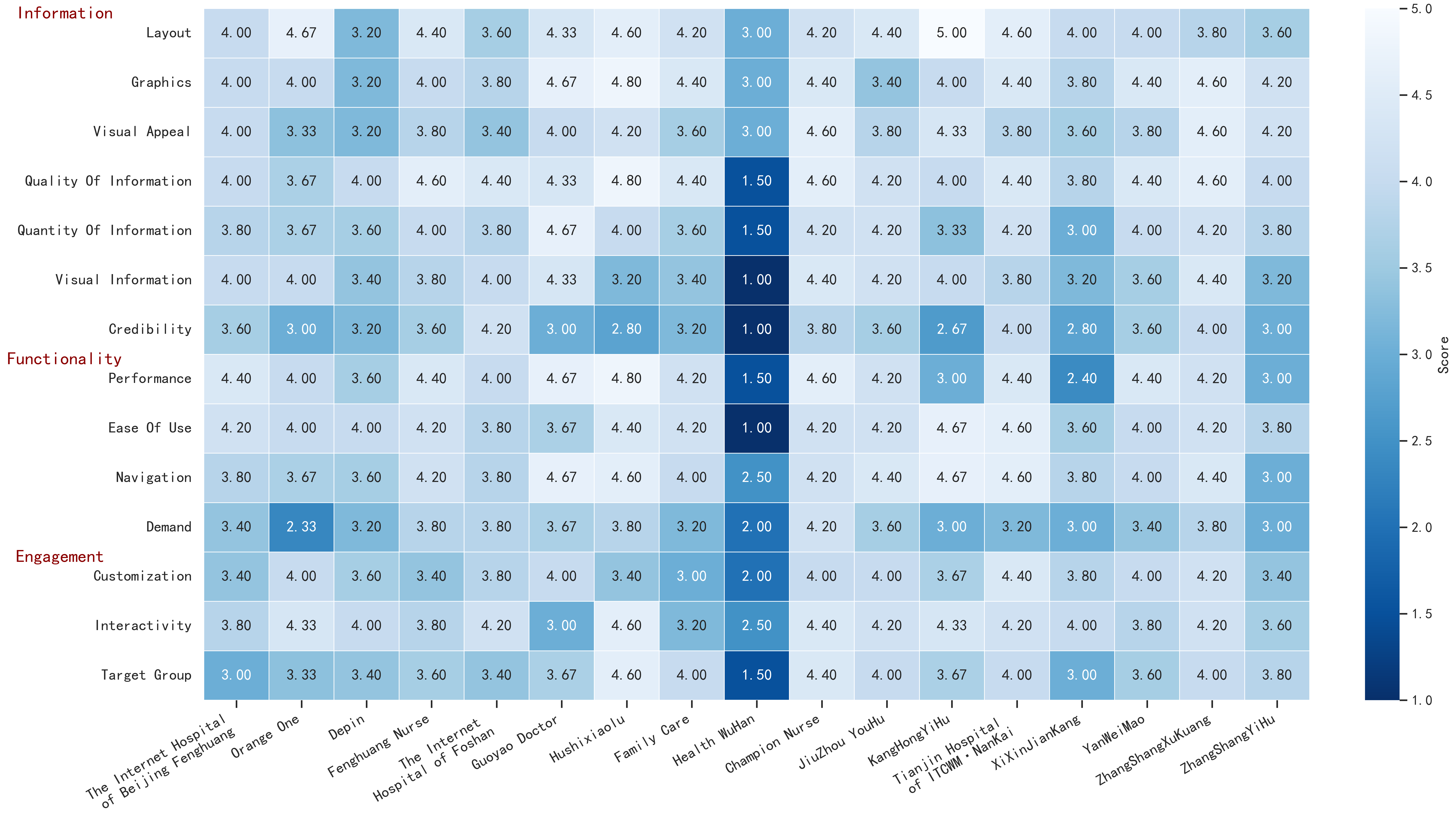

Supplement: Multimedia Appendix 2 [file mhealth-v12-e52169-s002.docx]
